# Autophagy-related protein 1 orchestrates autophagy initiation and feedback degradation in Alternaria alternata

**DOI:** 10.21203/rs.3.rs-8345089/v1

**Published:** 2026-02-11

**Authors:** Celine Yen Ling Choo, Hsin-Yu Lu, Kuang-Ren Chung, Pei-Ching Wu

**Affiliations:** National Chung Hsing University; National Chung Hsing University; National Chung Hsing University; China Medical University

**Keywords:** AIM, Peroxisome turnover, Nutrient sensing, Oxidative stress tolerance, Fungal pathogenicity, Autophagic flux regulation

## Abstract

**Background:**

Autophagy plays an essential role in fungal development and stress adaptation, yet its regulatory mechanisms in filamentous fungi remain incompletely understood. We functionally characterized *Alternaria alternata* Atg1 (AaAtg1), a serine/threonine kinase, and demonstrated its dual roles in autophagy initiation and flux modulation.

**Results:**

Deletion of *AaAtg1* abolishes autophagosome formation and autophagic flux, impairs peroxisome degradation, and leads to hypersensitivity to oxidative stress, as well as reduced virulence. AaAtg1 physically interacts with core autophagy proteins AaAtg13 and AaAtg8, and its vacuolar degradation is AaAtg8-dependent. Structure-guided mutagenesis of the Atg8-family interacting motif (AIM) disrupts AaAtg1–AaAtg8 binding in yeast two-hybrid assays but not in bimolecular fluorescence complementation, suggesting partial functional retention *in vivo*. Intriguingly, AIM mutations do not impair autophagy; instead, some transformants exhibit elevated autophagic activity, suggesting a potential negative regulatory role of AIM in autophagy tuning.

**Conclusions:**

These findings reveal a noncanonical feedback mechanism in which AaAtg8 facilitates AaAtg1 degradation to modulate autophagic output. Our study elucidates the structure–function relationship of AaAtg1 and uncovers a dual regulatory mechanism that coordinates autophagy progression and stress adaptation in the plant-pathogenic fungus.

## Introduction

Eukaryotic cells utilize autophagy, an intracellular degradation and recycling process, to maintain cellular homeostasis and adapt to environmental stress [1]. Autophagy is mediated by the coordinated action of an intricate machinery involving a group of autophagy-related proteins (Atgs) [2]. The process begins at the isolation membrane, where several Atg proteins are assembled to form a pre-autophagosomal structure (PAS) (also called phagophore assembly site) [2, 3]. A key player in this early step is Atg1, a serine/threonine kinase homologous to the mammalian Unc-51-like kinase 1 (ULK1) [4, 5]. In yeast, Atg1 forms a complex with Atg13 and associates with the Atg17-Atg31-Atg29 trimer to localize to the PAS and initiate autophagosome formation [6–9]. Subsequently, PAS expands to form double-membraned autophagosomes that engulf damaged proteins and organelles [8]. This self-recycling mechanism is crucial for cell survival under stressful conditions and for maintaining cellular homeostasis under ordinary circumstances. Autophagy is required for various cellular processes in filamentous fungi, including growth, cellular differentiation, development, conidiation, appressorium formation, and virulence [10–13]. While the importance of autophagy in nutrient limitation and stress responses is well recognized, its regulatory mechanisms, particularly the interplay among key autophagy proteins, remain incompletely understood in plant-pathogenic fungi.

Atg1 phosphorylation is regulated by several kinases, including the Target of Rapamycin kinase (TOR) [14, 15], the AMP-activated protein kinase (AMPK) [16], and protein kinase A (PKA) [17, 18]. Nutrient availability critically determines TOR activity, which in turn modulates Atg1 function. Under nutrient-poor conditions, TOR is suppressed, allowing Atg13 to remain dephosphorylated and to bind Atg1 with high affinity, thereby activating autophagy [19]. Conversely, under nutrient-rich conditions, TOR phosphorylates Atg13, weakening its interaction with Atg1 and inhibiting autophagy. The phosphorylated Atg1 phosphorylates Atg13 to regulate autophagy initiation and directly phosphorylates Atg11 to control selective autophagy processes such as pexophagy [19–21]. This dynamic phosphorylation-dephosphorylation cycle ensures precise regulation of autophagy in response to cellular nutrient status.

Autophagosome membrane expansion requires the ubiquitin-like protein Atg8 [22], which conjugates to phosphatidylethanolamine (PE) and anchors to the membrane surface [23]. In mammals, Atg8 homologs, such as microtubule-associated protein light chain 3 (LC3) or the gamma-aminobutyric acid A receptor-associated protein (GABARAP) [24], also serve as markers for monitoring autophagy formation [24, 25]. Beyond facilitating membrane expansion, the Atg8-PE conjugate plays key roles in cargo selection and autophagosome-vacuole fusion [26, 27]. It interacts with receptor proteins containing Atg8-family-interacting motifs (AIM) in yeast or LC3-interacting region (LIR) in mammals, thereby sequestering target proteins or damaged organelles during autophagosome expansion [28, 29]. Additionally, Atg8 recruits Atg1/ULK1 to autophagosomes, facilitating their maturation and fusion with vacuoles, a process crucial for degrading the Atg1/Atg13 complex [23, 29]. This interaction relies on the AIM of Atg1, which is essential for its binding to Atg8, as demonstrated in yeast. Mutations in the AIM disrupt Atg1-Atg8 binding, impair Atg1 transport to vacuoles, and hinder autophagy [27, 30].

*Alternaria alternata* is a necrotrophic fungal pathogen that causes diseases in more than 100 plant species and obtains nutrients exclusively from dead tissues [31]. Many *A. alternata* pathogens produce host-selective toxins, which are major contributors to pathogenicity [32]. During host colonization, *A. alternata* must also overcome the toxicity of reactive oxygen species (ROS) generated by the plant. We have previously shown that pexophagy, the selective autophagic degradation of peroxisomes, plays a crucial role in ROS resistance and cellular homeostasis in *A. alternata* [33, 34]. Moreover, our recent study revealed that autophagy regulates siderophore production and intracellular iron homeostasis, thereby contributing to ROS detoxification; notably, Δ*AaAtg1* failed to produce siderophores and displayed hypersensitivity to the iron chelator bathophenanthrolinedisulfonic acid (BPS) [35].

In this study, we sought further to dissect the role of AaAtg1 in stress-induced autophagy, focusing on its contributions to autophagic flux and broader regulatory functions. Our results indicate that AaAtg1 is essential for autophagy and that its degradation depends on interaction with AaAtg8. Intriguingly, mutations in the AIM of AaAtg1 disrupted its interaction with AaAtg8 but paradoxically enhanced autophagic activity in certain mutant strains. These observations point to a previously unrecognized dual role of AaAtg1 in both initiating and modulating autophagy, highlighting the complexity of autophagy regulation in filamentous fungi.

## Results

### AaAtg1 is required for growth and development

To investigate the role of *AaAtg1* in autophagy and pathogenesis in *A. alternata*, two deletion mutant strains (designated Δ*AaAtg1*-D6 and Δ*AaAtg1*-D7) were generated using a split-marker approach. Compared with the wild type, Δ*AaAtg1* exhibited slower growth, with 10% and 30% reductions in potato dextrose agar (PDA) and minimal medium (MM), respectively (Supplementary Figure S1A). Wild-type conidia were septated and ellipsoidal or cylindrical, whereas Δ*AaAtg1* produced slender conidia and abundant hyphal fragments (Supplementary Figure S1B, C). Germination of Δ*AaAtg1* conidia was markedly slower than that of the wild type. Additionally, Δ*AaAtg1* produced significantly fewer conidia and appressorium-like structures than the wild type (Supplementary Figure S1D, E). Genetic complementation of Δ*AaAtg1* with a functional *AaAtg1* copy (Cp8 strain) restored wild-type growth, conidiation, appressorium formation, and germination.

### AaAtg1 is required for autophagy

**Figure 1:**
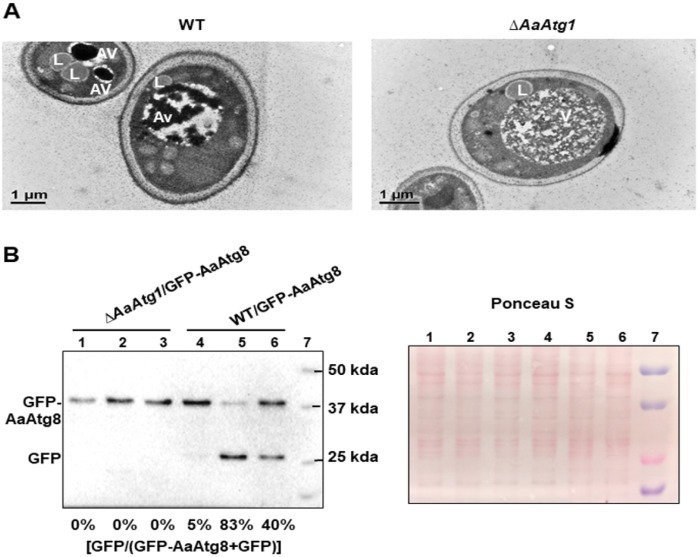
AaAtg1 is essential for autophagy. (**A**) TEM images of wild-type (WT) and Δ*AaAtg1* hyphae showing autophagic vacuoles (AV), lipid bodies (L), and vacuoles (V). (**B**) Western blot of GFP-AaAtg8 using anti-GFP antibody. Fungal strains were grown in PDB for 24 h and transferred to PDB, nitrogen-starvation medium (MM-N), or 15 mM H_2_O_2_ for 4 h. Autophagy activity was assessed by GFP-AaAtg8 cleavage. The percentage of free GFP was calculated from band intensities. Ponceau S staining confirms equal loading. Δ*AaAtg1*/GFP-AaAtg8 grown in PDB (Lane 1), MM-N (Lane 2), and H_2_O_2_ (Lane 3); WT/GFP-AaAtg8 grown in PDB (Lane 4), MM-N (Lane 5), and H_2_O_2_ (Lane 6); and protein marker (Lane 7).

Transmission electron microscopy (TEM) examination revealed that autophagic vacuoles (AVs) containing dense dark contents were present in the wild-type hyphae grown in MM-N (minimal medium without nitrogen) (Fig. 1A). In contrast, Δ*AaAtg1* lacked such AVs; most of them contained no dense contents. Western blot analyses revealed that Δ*AaAtg1* expressing GFP-AaAtg8 failed to initiate autophagy when exposed to hydrogen peroxide (H_2_O_2_) or MM-N, while the wild type accumulated free GFP, indicative of active autophagy (Fig. 1B).

**Figure 2:**
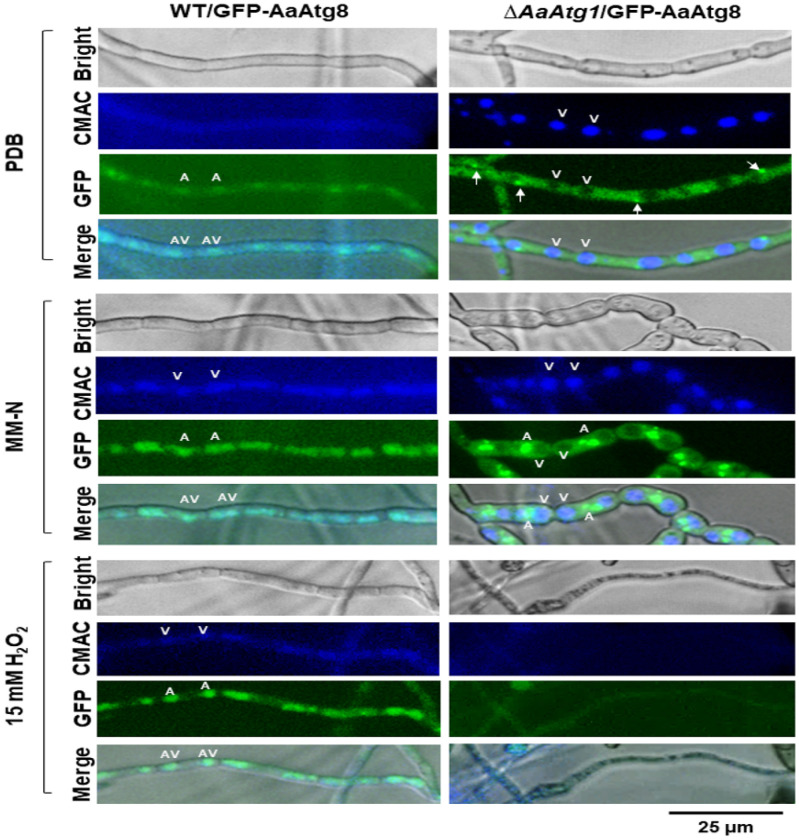
*AaAtg1* deficiency blocks the GFP-AaAtg8 transport into vacuoles. Microscopic observation of GFP-AaAtg8 localization in the hyphae from the wild-type strain (WT/GFP-AaAtg8) and the *AaAtg1*mutant (Δ*AaAtg1*/GFP-AaAtg8) strains grown in nutrient-rich conditions (PDB), nitrogen-starvation (MM-N), or 15 mM H_2_O_2_ for 24 h. Green fluorescence from GFP-AaAtg8 is detected to localize autophagosomes (A). Vacuoles (V) stained with CMAC dye, showing as faint blue spots. Co-localization of green and blue fluorescence indicates autophagic vacuole (AV) formation. In WT strains, autophagic vacuoles form under MM-N and H_2_O_2_ conditions, but not under PDB conditions. In Δ*AaAtg1* hyphae, autophagosomes are predominantly located outside vacuoles. Pre-autophagosomal structures (PAS, indicated by arrows) in the Δ*AaAtg1* hyphae grown in PDB. Scale bar = 25 μm.

Fluorescence microscopy further confirmed these findings. In wild-type hyphae incubated in MM-N or treated with H_2_O_2_ for 24 h, round blue fluorescent spots [7-amino-4-chloromethylcoumarin (CMAC)-stained vacuoles] were colocalized with green fluorescent spots from GFP-AaAtg8, indicating autophagosomes within vacuoles (Fig. 2). In contrast, in Δ*AaAtg1* hyphae cultured in MM-N, green fluorescent spots were observed near vacuoles but did not colocalize with them. When exposed to H_2_O_2_ for 24 h, Δ*AaAtg1* hyphae exhibited blurry green fluorescence, weak CMAC staining, and abnormal hyphal morphology, suggesting cell death.

### AaAtg1 is involved in peroxisome turnover

**Figure 3:**
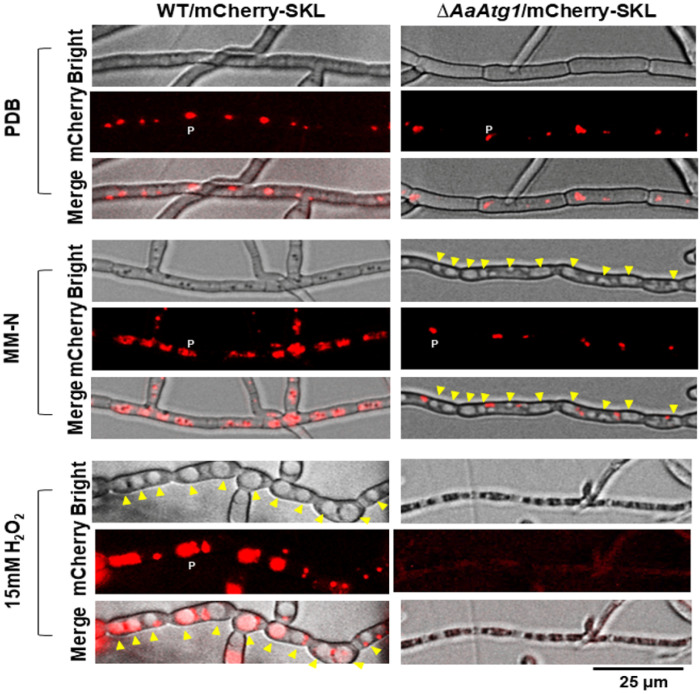
AaAtg1 is involved in the peroxisome degradation. Microscopic images of the wild-type (WT/mCherry-SKL) and the *AaAtg1*mutant (Δ*AaAtg1*/mCherry-SKL) strains expressing mCherry tagged with a serine-lysine-leucine (SKL) tripeptide at the carboxyl terminus to label peroxisomes (P). Fungal hyphae were grown in PDB, MM-N, or 15 mM H_2_O_2_ for 24 h and examined microscopically. Distinct red fluorescent spots indicate peroxisomes, and yellow arrowheads mark vacuoles within the hyphae. Co-localization of red fluorescent spots with vacuoles indicates pexophagy. In WT, pexophagy occurs in response to MM-N and H_2_O_2_, but not in PDB. In contrast, Δ*AaAtg1* fails to form pexophagy in all tested conditions. Scale bar = 25 μm.

To determine the role of AaAtg1 in pexophagy, a mCherry tag fused with serine-lysine-leucine (SKL) tripeptides was expressed in wild-type and Δ*AaAtg1* strains to label peroxisomes. Fluorescence microscopy revealed that in both WT/mCherry-SKL and Δ*AaAtg1*/mCherry-SKL strains, red fluorescence (representing peroxisomes) was not localized in vacuoles when cultured in potato dextrose broth (PDB) for 24 h. In the wild-type hyphae, after being shifted to MM-N or treated with H_2_O_2_, red fluorescence was observed in the vacuoles, indicating active pexophagy (Fig. 3). In contrast, red fluorescent spots were not localized in the vacuoles of Δ*AaAtg1*/mCherry-SKL hyphae cultured in MM-N, suggesting impaired pexophagy. Furthermore, under H_2_O_2_ treatment, Δ*AaAtg1*/mCherry-SKL hyphae exhibited deformed morphology, lacked distinct vacuoles, and showed no discernible red fluorescence, indicating severe cellular damage and disrupted peroxisomal turnover.

### AaAtg1 is involved in the detoxification of ROS

Due to the observed shrinkage and accumulation of dense materials in Δ*AaAtg1* hyphae exposed to H_2_O_2_, sensitivity tests were conducted on PDA amended with H_2_O_2_, *tert*-butyl hydroperoxide (tBHP), or cumyl peroxide. Compared to the wild type, Δ*AaAtg1* exhibited increased hypersensitivity to all three oxidants (Supplementary Figure S2A, B). However, Δ*AaAtg1* displayed wild-type sensitivity to single oxygen-producing compounds (rose Bengal and eosin Y) and cell wall stressors, sodium dodecyl sulfate (SDS), Congo red, and calcofluor white (data not shown). When treated with H_2_O_2_ and stained with 2’-7’-dichlorofluorescein diacetate (DCFHDA), Δ*AaAtg1* hyphae showed stronger green fluorescence, indicating greater ROS accumulation than the wild type (Supplementary Figure S2C). Similarly, propidium iodine (PI) staining revealed significantly stronger red fluorescence in Δ*AaAtg1* hyphae, indicating a higher proportion of dead cells after H_2_O_2_ treatment.

### AaAtg1 is required for full virulence but not for host-selective toxin production

Pathogenicity tests conducted on detached calamondin leaves revealed a significant reduction of necrotic lesions after inoculation with conidial suspensions of Δ*AaAtg1* 4 days post-inoculation (dpi) (Supplementary Figure S3A). In contrast, leaves inoculated with conidia from wild-type and CP8 strains developed necrotic lesions within the same period. Pre-wounding of leaves did not enhance lesion formation by the Δ*AaAtg1* strain. Microscopic examination revealed that conidia from wild-type and CP8 strains germinated rapidly and formed hyphae on leaf surfaces at 1 dpi (Supplementary Figure S3B). However, conidia from Δ*AaAtg1* showed delayed germination on leaves until 2–3 dpi (data not shown), correlating with the delayed necrotic lesion development. Thin-layer chromatography (TLC) and High-performance liquid chromatography (HPLC) analyses revealed no quantitative difference in host-selective ACT toxin production between wild-type and Δ*AaAtg1* strains (Supplementary Figure S4A, B).

### AaAtg1 physically interacts with both AaAtg13 and AaAtg8 in A. alternate

**Figure 4:**
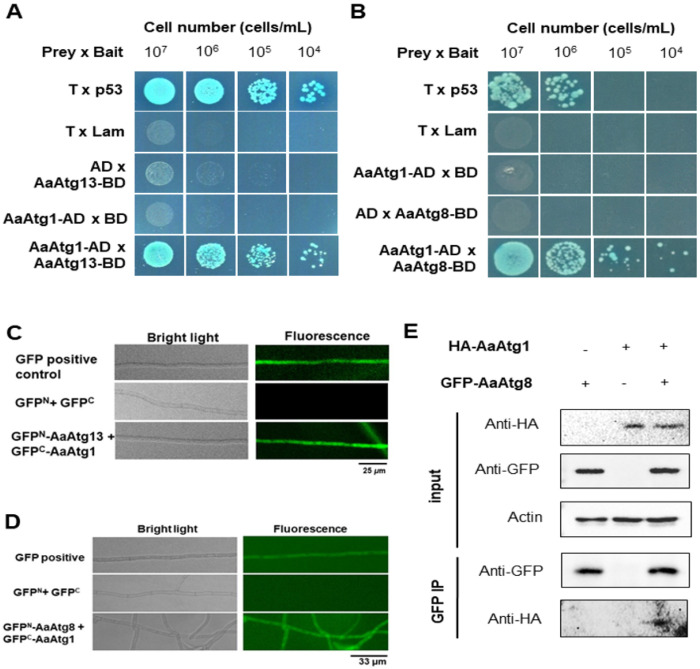
AaAtg1 physically interacts with AaAtg13 and AaAtg8. (**A**) (**B**) Y2H assays. Yeast cells expressing AaAtg1-AD (prey) and AaAtg13-BD or AaAtg8-BD (Bait) successfully grew on QDO/X/A plates, indicating interaction. Positive control (T-prey × p53-bait) also supported growth, while negative controls (T-prey × Lam-bait, AaAtg1-AD × BD-empty, and AD-empty × AaAtg13-BD/AaAtg8-BD) showed no growth. (**C**) (**D**) BiFC assays. Co-expression of GFP^N^-AaAtg13 or GFP^N^-AaAtg8 with GFP^C^-AaAtg1 in wild-type protoplasts produced green fluorescence, confirming interactions *in vivo*. Co-expression of GFP^N^ and GFP^C^ (negative control) yielded no fluorescence, while full-length GFP (positive control) displayed green fluorescence. (**E**) In Co-IP assays, strains co-expressing AaAtg1-HA and GFP-AaAtg8 were cultured in PDB for 24 h. Immunoprecipitation with GFP-Trap Agarose, followed by immunoblotting using anti-GFP and anti-HA antibodies, confirmed the interaction. Actin was used as a loading control.

To determine whether AaAtg1 physically interacts with other core autophagy components, we performed Yeast two-hybrid (Y2H) assays using AaAtg13 and AaAtg8 as potential binding partners. Yeast cells expressing AaAtg1 fused to the GAL4 activation domain (AaAtg1-AD) and either AaAtg13 or AaAtg8 fused to the binding domain (AaAtg13-BD, AaAtg8-BD) thrived on QDO/X/A selective medium (Fig. 4A, B), indicating direct interactions between AaAtg1 and both proteins. These interactions were further validated *in vivo* using bimolecular fluorescence complementation (BiFC) assays. Co-expression of GFP^N^-AaAtg13 with GFP^C^-AaAtg1, or GFP^N^-AaAtg8 with GFP^C^-AaAtg1, in *A. alternata* protoplasts led to the appearance of green fluorescent signals along the hyphal compartments (Fig. 4C, D), while the negative control strains expressing only split GFP fragments showed no fluorescence, confirming the specificity of these interactions. To further confirm the interaction between AaAtg1 and AaAtg8 under native conditions, co-immunoprecipitation (Co-IP) was performed. Cell lysates from *A. alternata* strains co-expressing HA-tagged AaAtg1 and GFP-tagged AaAtg8 were immunoprecipitated using anti-GFP antibodies. Western blotting detected HA-AaAtg1 in the precipitated complex, confirming its association with GFP-AaAtg8 *in vivo* (Fig. 4E). Together, AaAtg1 physically interacted with both AaAtg13 and AaAtg8, supporting its central role as a scaffold protein coordinating autophagy-related protein assembly in *A. alternata*.

### The AIM region affects the AaAtg1 structure and its interaction with AaAtg8

Amino acid sequence analysis revealed that AaAtg1 possesses several conserved domains associated with autophagy regulation. At the N-terminus, AaAtg1 contains a canonical serine/threonine kinase domain. In contrast, its C-terminal region harbors two microtubule-interacting and transport (MIT) domains, which are predicted to mediate interaction with AaAtg13 (Supplementary Figure S5A). Additionally, sequence inspection identified a putative AIM/LIR, characterized by the conserved sequence EGFVFVEK, located between the kinase and MIT domains (Supplementary Figure S5B). This motif could likely serve as a docking site for AaAtg8 and is conserved among Atg1 orthologs in other filamentous fungi.

**Figure 5:**
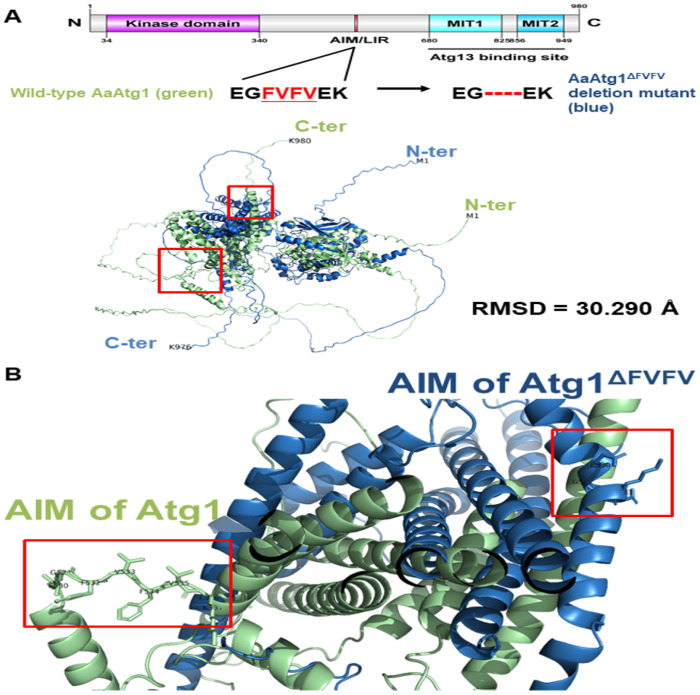
Structural impact of AIM deletion on AaAtg1 conformation predicted by AlphaFold2. (**A**) Schematic representation of AaAtg1 protein domains, including the kinase domain, MIT motifs, Atg13-binding region, and AIM/LIR region (EGFVFVEK). Structural models of wild-type AaAtg1 (green) and the AIM-deletion variant AaAtg1^ΔFVFV^ (blue; residues 532–535 removed) were generated using AlphaFold2. Superimposition of the two models reveals a substantial overall conformational shift, with a root-mean-square deviation (RMSD) of 30.290, indicating that deletion of the AIM region causes widespread structural rearrangement. (**B**) Enlarged view of the AIM region (highlighted in red boxes). In the wild-type protein, the AIM adopts a defined orientation and side-chain positioning. In contrast, deletion of FVFV residues in AaAtg1^ΔFVFV^ leads to significant distortion of the local helical structure, suggesting a loss of structural integrity required for stable Atg8 binding.

**Figure 6:**
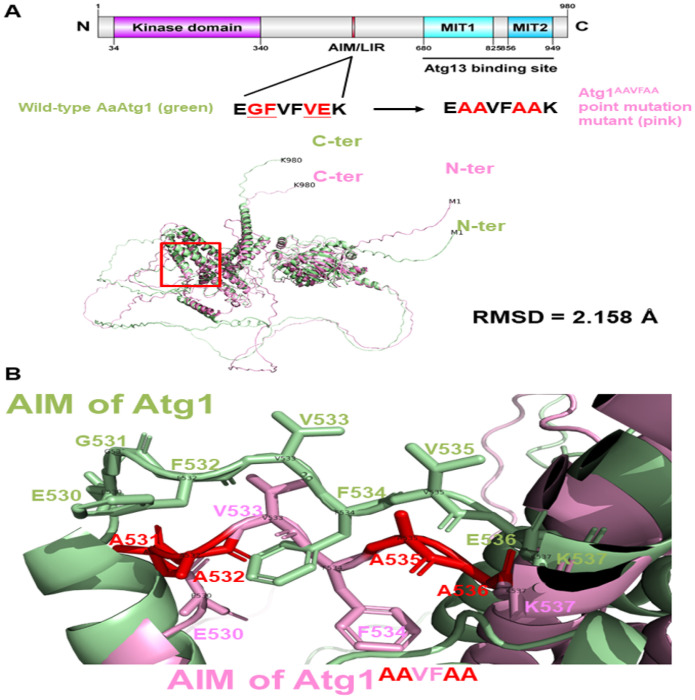
Structural modeling of wild-type AaAtg1 and its AIM point-mutant variant. (**A**) AlphaFold2-generated structural models of wild-type AaAtg1 (green, residues 530–537: EGFVFVEK) and the AaAtg1^AAVFAA^ point-mutant (pink, EAAVFAAK), which replaces the core AIM residues G531, F532, V535, and E536 with alanine. Superimposition reveals an RMSD of 2.158, suggesting a modest global structural deviation. (**B**) Magnified view of the AIM region shows local conformational shifts in the AaAtg1 ^AAVFAA^ mutant. Red-highlighted side chains represent the alanine substitutions. Despite overall structural preservation, mutations in AIM alter residue positioning, potentially affecting Atg8 docking and downstream autophagy regulation.

To evaluate the structural impact of the AIM region in AaAtg1, we used AlphaFold2 to model two variants: a deletion mutant (ΔFVFV; residues 532–535 removed) and a point-mutated version (AAVFAA; EGFVFVEK → EAAVFAAK). Superimposition with the wild-type model revealed that the ΔFVFV deletion induced a substantial global structural rearrangement (RMSD = 30.290) (Fig. 5A). In contrast, the AAVFAA substitution caused only minor overall changes (RMSD = 2.158) (Fig. 6A). Nonetheless, both mutants exhibited pronounced local alterations at the AIM region, notably affecting loop structure and side-chain positioning (Figs. 5B and 6B).

**Figure 7:**
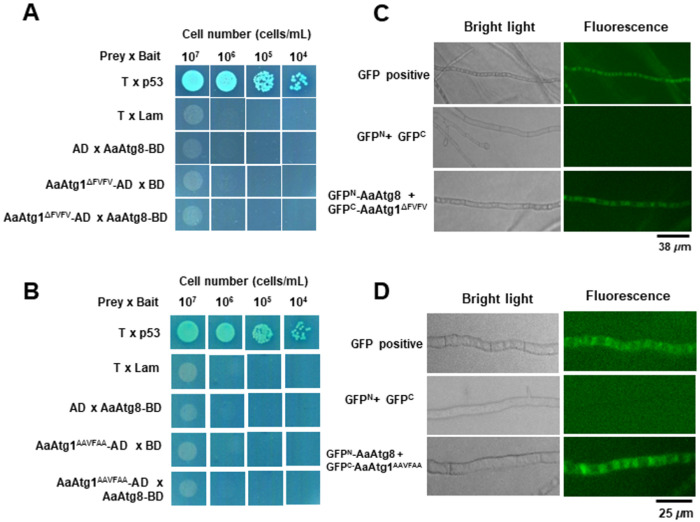
Deletion or mutation of the AIM region in AaAtg1 disrupts interaction with AaAtg8. (**A**) Y2H assays show that yeast cells expressing AaAtg1^ΔFVFV^-AD (prey) and AaAtg8-BD (bait) failed to grow on QDO/X/A plates, indicating a disrupted interaction. Positive control (T-prey × p53-bait) supports growth, while negative controls (T-prey × Lam-bait, AaAtg1ΔFVFV-AD × BD-empty, and AD-empty × AaAtg8-BD) show no growth. (**B**) Y2H assays. Yeast cells expressing AaAtg1^AAVFAA^-AD (prey) and AaAtg8-BD (bait) fail to grow on QDO/X/A plates, confirming disrupted interaction. (**C**) BiFC assays. Co-expression of GFP^N^-AaAtg8 and GFP^C^-AaAtg1^ΔFVFV^ in wild-type protoplasts results in green fluorescence, confirming the presence of a residual interaction. Co-expression of GFP^N^ and GFP^C^ (negative control) yields no fluorescence. (**D**) BiFC assays. Co-expression of GFP^N^-AaAtg8 and GFP^C^-AaAtg1AAVFAA in wild-type protoplasts results in green fluorescence, indicating a retained but altered interaction *in vivo*.

To investigate whether these structural changes disrupt protein–protein interactions, we performed Y2H and BiFC assays. In Y2H, neither the ΔFVFV nor AAVFAA variants showed detectable interaction with AaAtg8 (Fig. 7A, B), suggesting reduced binding affinity *in vitro*. However, BiFC assays revealed green fluorescence in both mutant strains (Fig. 7C, D), indicating residual or altered interaction *in vivo*.

### The AIM mutation could increase autophagy under certain conditions

**Figure 8:**
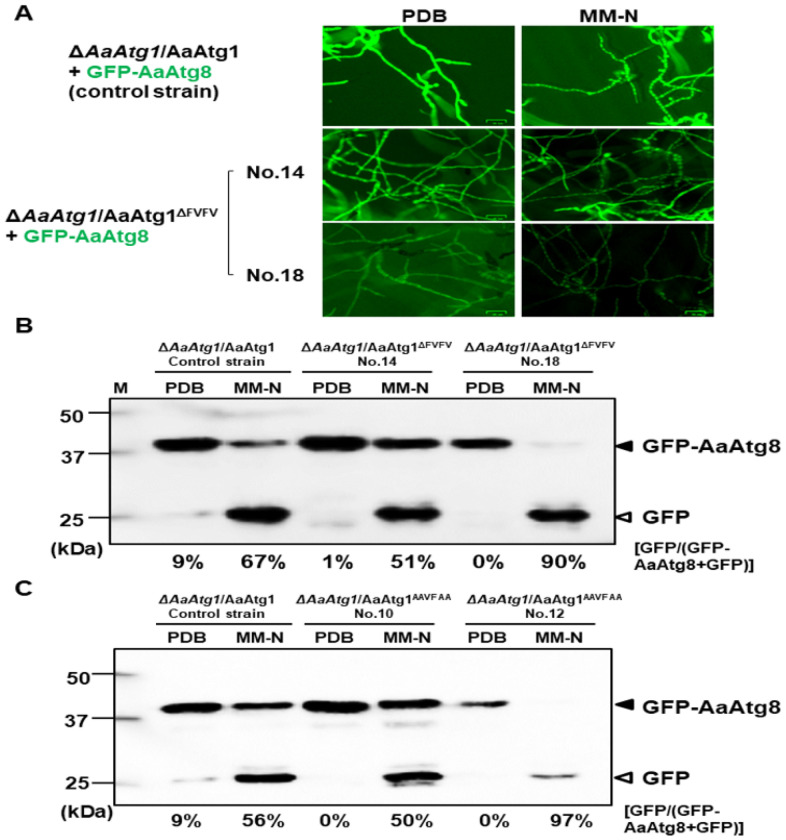
The AIM region of AaAtg1 modulates autophagy in *A. alternata*. (**A**) Microscopic images showing green fluorescence from the Δ*AaAtg1* co-expressing AaAtg1 and GFP-AaAtg8 (control strain). Δ*AaAtg1* co-expressing AaAtg1^ΔFVFV^ and GFP-AaAtg8 resulted in two types of transformants. One group (14 transformants, exemplified by No. 14) exhibits fluorescence patterns and intensities similar to the control strain. The other group (18 transformants, exemplified by No. 18) displays weak fluorescence spots within the hyphae. Fungal strains were grown in PDB overnight and shifted to MM-N for 6 h. Scale bar = 25 μm. (**B**) Western blot analysis of GFP-AaAtg8 cleavage in the control strain, transformant No. 14, and transformant No.18. The cleavage of GFP-AaAtg8 indicates autophagic activity. (C) Western blot analysis of GFP-AaAtg8 cleavage in the control strain and Δ*AaAtg1* transformants (No. 10 and No. 12) co-expressing AaAtg1^AAVFAA^ and GFP-AaAtg8. Fungal hyphae grown in PDB overnight were harvested, washed with sterile water, and shifted to MM-N for 6 h.

To investigate the role of the AIM in autophagy flux, Δ*AaAtg1* co-expressing AaAtg1^ΔFVFV^ (deletion) or AaAtg1^AAVFAA^ (point mutation) with GFP-AaAtg8 were further examined. The control strain, Δ*AaAtg1* co-expressing wild-type AaAtg1 and GFP-AaAtg8, displayed uniform green fluorescence in hyphae grown in PDB and distinct fluorescent spots after being shifted to MM-N (Fig. 8A). Δ*AaAtg1*/AaAtg1^ΔFVFV^ transformants exhibited two distinct fluorescent patterns, approximately in a 1:1 ratio. One group (14 transformants), exemplified by transformant No. 14, exhibited green fluorescent patterns and intensities similar to those of the control strain. The other group (18 transformants), represented by transformant No. 18, displayed weak fluorescent spots under MM-N. Western blot analysis revealed that free GFP cleavage from GFP-AaAtg8 was 51% in transformant No. 14, comparable to the control strain (67%) (Fig. 8B). However, transformant No. 18 exhibited nearly 90% of free GFP under MM-N conditions, indicating enhanced autophagic flux. Similarly, Δ*AaAtg1*/AaAtg1^AAVFAA^ transformants also showed two distinct fluorescent patterns. Transformant No. 10 displayed strong fluorescence comparable to the control strain, while other transformant No. 12 exhibited weak fluorescence (data not shown). Western blot analysis confirmed ~ 50% free GFP in transformant No. 10, similar to the control, but ~ 97% free GFP in transformant No. 12, further supporting enhanced autophagy when the AIM region was mutated (Fig. 8C).

### Localization of AaAtg1 to vacuoles is triggered by nitrogen starvation and mediated by AaAtg8

**Figure 9:**
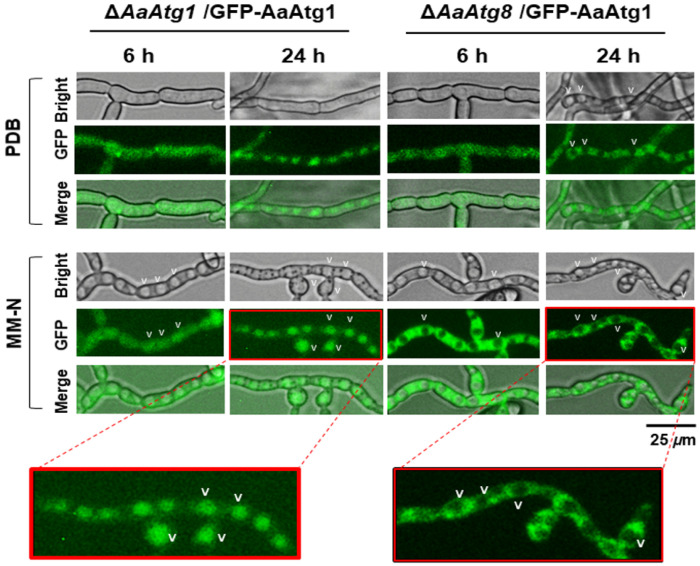
AaAtg8 mediates the recruitment and degradation of AaAtg1 in vacuoles. Microscopic images showing green fluorescence emitted by GFP-AaAtg1 in the Δ*AaAtg1* and the Δ*AaAtg8*strains. Conidia were grown in PDB overnight, shifted to MM-N, and incubated for 6 and 24 h. GFP-AaAtg1 is localized to vacuoles in Δ*AaAtg1* under nitrogen starvation, while its localization is disrupted in Δ*AaAtg8*, indicating the role of AaAtg8 in facilitating AaAtg1 degradation in vacuoles. Scale bar = 25 μm.

To investigate the localization of AaAtg1 during autophagy and the role of AaAtg8 in this process, GFP-AaAtg1 was expressed in Δ*AaAtg1* and Δ*AaAtg8* strains, and the resulting transformants were examined microscopically (Fig. 9). In Δ*AaAtg1*/GFP-AaAtg1 hyphae (complementation strain), green fluorescence was uniform after being shifted to PDB for 6 h, with no visible vacuoles. Following 6 h in MM-N, vacuoles became visible, but green fluorescence was barely detectable within them. After 24 h in PDB or MM-N, green fluorescence formed distinct patches, suggesting vacuolar localization during prolonged starvation. In contrast, Δ*AaAtg8*/GFP-AaAtg1 hyphae emitted uniform green fluorescence with no visible vacuoles after 6 h in PDB. At 24 h, vacuoles became visible, but green fluorescence was excluded from vacuoles. Similarly, after 6 or 24 h in MM-N, vacuoles in Δ*AaAtg8*/GFP-AaAtg1 hyphae contained no green fluorescence, indicating the requirement of AaAtg8 for the proper localization of AaAtg1 to vacuoles during autophagy.

## Discussion

The necrotrophic pathogen *A. alternata* produces host-selective toxins [36] and cell wall-degrading enzymes [37] to kill its host plant, leading to the accumulation of toxic ROS [38]. *A. alternata* must detoxify ROS to obtain nutrients from dead cells [39]. Current studies have shown the important role of AaAtg1 in ROS- and starvation-sensing, regulating autophagy initiation and cargo degradation. AaAtg1 is required for normal autophagy formation, which plays a crucial role in growth, conidiation, conidial germination, appressorial development, and pathogenesis. AaAtg1 is also responsible for autophagy-mediated peroxisome turnover. AaAtg1-mediated autophagy formation plays an important role in cellular resistance to ROS and cell survival under oxidative stress and starvation. AaAtg1 physically interacts with AaAtg8 and AaAtg13 to initiate autophagy, and AaAtg8 is required for AaAtg1 degradation in the vacuoles. We have further demonstrated that the AIM region of AaAtg1 is required for binding to AaAtg8 in Y2H but not in BiFC assays. Surprisingly, mutating the AIM region did not affect autophagy formation in some transformants but did in others. This suggests a leaky regulation in autophagy formation once the AIM region is mutated.

In both yeast and mammals, the Atg1/ULK1 complex integrates nutrient starvation signals and recruits core autophagy proteins to the PAS to initiate autophagy [5]. However, in *A. alternata*, deletion of AaAtg1 does not abolish PAS-like structures. As shown in Fig. 2, GFP-AaAtg8 puncta remain detectable under nutrient-rich conditions, appearing as small bright fluorescent dots, suggesting that AaAtg1 is not essential for the basal recruitment of Atg8 to the PAS. Under nitrogen starvation, larger GFP-AaAtg8 puncta accumulate adjacent to vacuoles in the Δ*AaAtg1* mutant but fail to enter them, and show no GFP cleavage. These results indicate a complete block in autophagic flux once AaATg1 is impaired. This phenotype partially resembles that of *Saccharomyces cerevisiae*, in which Atg1 is dispensable for the initial PAS localization of Atg8 and Atg9 but is required for autophagosome formation and progression of autophagy [6, 40]. In *A. alternata*, however, AaAtg1 appears to play a more stringent role. Although PAS-like puncta can form without it, subsequent steps, including autophagosome maturation, vacuolar fusion, and cargo degradation, are completely impaired. This strict dependency is corroborated by TEM and proteolytic assays, which confirm the absence of autophagic vacuoles in Δ*AaAtg1* under both nitrogen starvation and oxidative stress.

Δ*AaAtg1* is highly sensitive to H_2_O_2_ and other peroxides. Although PAS and autophagosomes are present in Δ*AaAtg1* hyphae under nutrient-poor conditions (e.g., MM-N), these structures are absent following H_2_O_2_ treatment, likely due to ROS-induced cell death. In contrast, wild-type hyphae form autophagic vacuoles under both nitrogen starvation and oxidative stress. DCFHDA staining reveals elevated intracellular H_2_O_2_ accumulation in Δ*AaAtg1* compared to the wild type, and PI staining confirms extensive cell death after H_2_O_2_ exposure. These findings underscore the importance of autophagy in maintaining redox homeostasis and promoting survival under oxidative stress. Previous studies show that H_2_O_2_ enhances peroxisome turnover in *A. alternata*, leading to decreased peroxisome levels [34]. We therefore investigated whether AaAtg1-mediated autophagy contributes to peroxisome degradation. Both nitrogen starvation and H_2_O_2_ induce peroxisome aggregation and translocation into vacuoles in the wild type (Fig. 3). In Δ*AaAtg1*, however, peroxisomes fail to undergo these changes: under MM-N, they remain scattered outside vacuoles, and after H_2_O_2_ exposure, they become undetectable. Given that peroxisomes are significant sites of H_2_O_2_ production [41], and defects in peroxisome biogenesis render fungal cells ROS-sensitive [42], the failure to degrade peroxisomes likely contributes to ROS accumulation and cell death in Δ*AaAtg1*. Together, these results highlight the essential role of AaAtg1 in pexophagy and oxidative stress resistance.

In yeast, Atg1 interacts with Atg8 in an AIM-dependent manner [27, 30]. The AIM region (EGFVFVEK) of AaAtg1 serves as a critical docking site for AaAtg8, as demonstrated by Y2H and BiFC assays. Y2H assays show that AIM mutations (ΔFVFV or EAAVFAAK) disrupt AaAtg8 binding, whereas BiFC assays reveal residual *in vivo* interaction, suggesting that the cellular context may enable weak or transient interactions. This points to a subtle regulatory mechanism of autophagy in *A. alternata*. Unexpectedly, AIM mutations do not uniformly suppress autophagy. Compared to wild-type, some transformants exhibit increased autophagic flux, as evidenced by stronger GFP-AaAtg8 cleavage. These findings imply that impaired AaAtg1–AaAtg8 interaction may reduce AaAtg1 degradation, leading to kinase stabilization and excessive autophagy activation. This “leaky regulation” contrasts sharply with yeast, where AIM mutations consistently suppress autophagy [30]. Together, our data suggest that the AIM region in *A. alternata* serves not only as a docking site but also as a gatekeeper that modulates autophagic intensity, highlighting a distinct regulatory mechanism that prevents detrimental hyperactivation.

Localization studies reveal that AaAtg1 translocates to vacuoles under nitrogen starvation, a process that is dependent on AaAtg8. In Δ*AaAtg8* hyphae, GFP-AaAtg1 fails to accumulate in vacuoles, indicating that AaAtg8 mediates the vacuolar sequestration and degradation of AaAtg1 during autophagy. This AaAtg8-dependent turnover likely functions as a feedback mechanism that downregulates autophagic activity and maintains homeostasis during prolonged stress. This observation aligns with findings in *S. cerevisiae*, where Atg1 undergoes degradation via an AIM/LIR–dependent mechanism [23]. Autophagy deficiency in Δ*AaAtg1* correlated with marked virulence attenuation, likely due to combined effects on hyphal growth, sporulation, and ROS detoxification. In contrast, AIM mutations did not impair virulence (data not shown), yet they revealed a possible inhibitory role of the AIM-mediated interaction in restricting excessive autophagy. This suggests that the AIM region not only facilitates Atg8 binding but also serves as a checkpoint to prevent overactivation of the autophagic pathway.

## Conclusion

This study demonstrates that AaAtg1 functions not only as a trigger for autophagy initiation but also as a substrate of autophagic degradation, thus constituting a negative feedback loop, a concept rarely described in plant-pathogenic fungi. The paradoxical enhancement of autophagic flux in AIM mutants may reflect the loss of this regulatory brake, a mechanism warranting further investigation. Together, these studies highlight AaAtg1 as a macromolecular scaffold that coordinates protein–protein interactions, signal transduction, and autophagic flux regulation in *A. alternata*. These insights expand our understanding of fungal stress adaptation and virulence control via autophagy, offering new perspectives on the molecular mechanisms that govern autophagy homeostasis in filamentous plant pathogens.

## Materials and Methods

### Fungal strains, conidiation, germination, and sensitivity assays

The wild-type strain of *A. alternata* used for transformation and mutagenesis has been previously characterized [43]. Fungal strains were point-inoculated onto PDA (Difco, Franklin Lakes, NJ, U.S.A.) or MM [44] agar using a sterile toothpick and incubated at 28°C. For conidiation, fungal strains were grown on PDA without parafilm sealing under light for 5 days. Conidia were collected in sterile water, and germination was assessed by incubating conidia on glass slides or 96-well microtiter plates at 28°C for 6 h.

Fungal sensitivity assays were conducted on PDA plates (90 mm × 15 mm Petri dishes) containing test compounds, including calcôuor white (200 μM in dimethyl sulfoxide), Congo red (75 μM in ethanol), sodium dodecyl sulfate (SDS, 0.01%), rose Bengal (30 μM), eosin Y (100 μM), hydroperoxide (H_2_O_2_, 20 mM), *tert*-butyl hydroperoxide (tBHP, 3.75 mM), and cumyl hydroperoxide (3 mM in ethanol). Unless otherwise specified, all compounds were dissolved in water. Each treatment was performed in triplicate, and experiments were independently repeated three times.

#### Genetic modification in A. alternata

The *AaAtg1* sequence (accession number: KAH8628380) was retrieved from the complete genome of the *A. alternata* EV-MIL-31 strain. Functional motifs within AaAtg1 were identified using the InterPro database and protein domain analysis tools [45]. A multiple sequence alignment illustrating the functional domains of the Atg1 homologs across different species was generated using the Illustrator for Biological Sequences (IBS) software [46]. All Atg1 protein sequences were obtained from the National Center for Biotechnology Information (NCBI) database (http://www.ncbi.nlm.nih.gov). The AIM/LIR region was predicted using the iLIR server (https://ilir.warwick.ac.uk/) [28]. Structure models of full-length AaAtg1 and its AIM-mutated variants were constructed using AlphaFold2 (https://colab.research.google.com/github/sokrypton/ColabFold/blob/main/AlphaFold2.ipynb) [47].

Two Δ*AaAtg1* mutants (D6 and D7) and a complementation strain (CP8) were previously generated and characterized [35]. Δ*AaAtg1* mutants were transformed with pCB1532-GFP-AaAtg8 or pCB1532-mCherry-SKL to generate Δ*AaAtg1*/GFP-AaAtg8 and Δ*AaAtg1*/mCherry-SKL strains, respectively, using protoplasts from the Δ*AaAtg1* D6 mutant. Protoplast preparation and fungal transformations were conducted as described previously [48]. Wild-type strains expressing GFP-AaAtg8 (WT/GFP-AaAtg8) or mCherry-SKL (WT/mCherry-SKL) from previous studies [33, 34] were used as controls. To investigate the localization of AaAtg1 in the absence of AaAtg8, the pNEOGPE1-GFP-AaAtg1 plasmid was constructed and transformed into protoplasts of both Δ*AaAtg1* and Δ*AaAtg8* strains, with the latter generated in a previous study [34]. Oligonucleotide primers used in this study are listed in Supplementary Table S1, and all plasmids are detailed in Supplementary Table S2.

### Site-directed mutagenesis and plasmid construction

Fungal strains were cultured in a liquid complete medium [49] on a shaker at 28°C for 2 days. Mycelium was harvested and ground in liquid nitrogen for RNA isolation using TRI-reagent (Sigma-Aldrich, St. Louis, MO, U.S.A.) and the PureLink RNA Kit (Invitrogen, Waltham, MA, U.S.A.) according to the manufacturer’s protocols. Complementary DNA (cDNA) was synthesized from the isolated RNA using iScript Reverse Transcriptase (Bio-Rad, Hercules, CA, U.S.A.).

Two-step fusion PCR mutagenesis was performed using cDNA as a template to generate the AaAtg1 AIM deletion (AaAtg1^ΔFVFV^) and point mutation (AaAtg1^AAVFAA^) variants. In the first step, two overlapping cDNA fragments were amplified using specific primers (Supplementary Table S1). These fragments were then used as templates for a second round of PCR to generate the full-length AaAtg1^ΔFVFV^ or AaAtg1^AAVFAA^ fragment. PCR products were digested with EcoRV and KpnI and cloned into the pCB1532 vector to yield pCB1532-AaAtg1^ΔFVFV^ or pCB1532-AaAtg1^AAVFAA^. The identity of the cloned cDNA fragments was confirmed by sequencing. Plasmids were individually transformed into protoplasts prepared from the Δ*AaAtg1* D6 mutant strain. The resulting strains were used for AaAtg8-AaAtg1 AIM binding studies. To assess the effect of AIM mutations on GFP-AaAtg8 expression and autophagy formation, a pBARS-GFP-AaAtg8 plasmid containing a bialaphos resistance gene was transformed into protoplasts prepared from the Δ*AaAtg1*/AaAtg1^ΔFVFV^ or Δ*AaAtg1*/AaAtg1^AAVFAA^ strain.

Fungal transformants were recovered on regeneration media supplemented with the appropriate selective agents, including bialaphos (400 μg/ml, FUJIFILM Wako Pure Chemical Corporation, Osaka, Japan), geneticin (G418, 300 μg/mL, Amresco, Solon, OH, U.S.A.), or sulfonylurea (10 μg/ml, ChemService Inc., West Chester, PA, U.S.A.). Fungal strains were further screened and validated by PCR using specific primers (Supplementary Table S1).

### Western blot analysis

Western blot analysis was performed as previously described [34]. Briefly, fungal strains were cultured in potato dextrose broth (PDB, Difco) for 24 h, then transferred to PDB, MM-N (MM without nitrogen), or 15 mM H_2_O_2_, and incubated for 4 h. Total proteins were extracted from mycelium using RIPA cell lysis buffer containing 50 mM Tris-HCl (pH 7.4), 150 mM NaCl, 1 mM EDTA, 0.1% Triton X-100, and 1% protease inhibitor cocktail (Biokit, Toufen City, Miaoli, Taiwan). Proteins were boiled in a loading buffer, separated by SDS-PAGE, and transferred onto PVDF membranes. Membranes were stained with Ponceau S to verify the transfer, blocked with Towbin’s (TSW) buffer containing 25 mM Tris, 192 mM Glycine, and often 20% methanol and 0.1% SDS, and incubated overnight at 4°C with a rabbit anti-GFP antibody (1:5000). After washing, membranes were incubated with HRP-conjugated goat anti-rabbit IgG (1:10,000, Jackson ImmunoResearch, West Grove, PA, U.S.A.). Signals were detected using LumiFlash Prime Chemiluminescent Substrate, HRP (Visual Protein, Taipei, Taiwan). Chemiluminescence images were captured with the ChemiDoc MP imaging system (Bio-Rad) and analyzed using ImageLab software (version 6.1.0, Bio-Rad). Band intensity was quantified using ImageJ software (US National Institutes of Health, Bethesda, MD, U.S.A.) (https://imagej.net/ij/).

### Co-immunoprecipitation (Co-IP)

For Co-IP experiments, an *AaAtg1* fragment was amplified using primers ATG1 F EcoRV and ATG1 R KpnI, digested with EcoRV and KpnI, and cloned into the pNEOGPE1-HA plasmid containing a linker HA-tag sequence (5’-atgggcagctacccatacgatgttccagattacgct-3’) to generate pNEOGPE1-HA-AaAtg1. The plasmids pNEOGPE1-HA-AaAtg1 and pCB1532-GFP-AaAtg8 were individually or co-transformed into wild-type protoplasts, resulting in three distinct fungal strains. Fungal strains were cultured in PDB for 24 h, and total proteins were extracted using RIPA buffer amended with Ser/Thr phosphatase inhibitors (1.0 mM Na_3_VO_4_ and 1.0 mM/l NaF; Sigma-Aldrich). Immunoprecipitation was performed using GFP-Trap Agarose beads (Chromotek, Islandia, NY, U.S.A.) according to the manufacturer’s instructions. Proteins were eluted from magnetic beads using 2× Laemmli buffer, and 25 μl of the sample was subjected to western blot analysis. Actin served as the internal control.

### Yeast two-hybrid (Y2H) assays

Y2H assays were conducted using the GAL4-based Matchmaker Yeast Two-Hybrid System (Clontech Laboratories, Mountain View, CA, U.S.A.). Full-length coding regions of AaAtg1, AaAtg1^ΔFVFV^, and AaAtg1^AAVFAA^ were individually amplified, fused with GAL4 activation domain (AD), and cloned into the pGADT7 plasmid to generate preys (AaAtg1-AD, AaAtg1^ΔFVFV^-AD, and AaAtg1^AAVFAA^-AD). Similarly, the full-length coding regions of AaAtg8 and AaAtg13 were fused to the GAL4 DNA-binding domain (DBD) and cloned into the pGBKT7 vector as baits (AaAtg8-BD and AaAtg13-BD).

Plasmids were propagated in *Escherichia coli* and introduced into the yeast Y187 or Y2HGold strains according to the manufacturer’s protocol (Clontech). Negative (T-prey x Lam-bait) and positive (T-prey x p53-bait) controls were included to validate the assay. Four independent transformants for each experimental pairing were tested. Yeast strains were plated on selective SD/–Leu/–Trp/–Ade/–His agar plates containing X-α-Gal (40 μg/ml) and Aureobasidin A (200 ng/ml) (QDO/X/A). Growth on the selective medium indicated a strong interaction between the tested proteins. Primers used for plasmid construction are listed in Supplementary Table S1, with restriction enzyme recognition sites incorporated to facilitate cloning.

### Bimolecular fluorescence complementation (BiFC) assay

The N-terminal half of GFP (GFP^N^) was amplified from pCB1532-GFP-AaAtg8 by PCR and subcloned into pHygroGPE1 to yield pHygroGPE1-GFP^N^. The C-terminal half of GFP (GFP^c^) was cloned into pNEOGPE1 to result in pNEOGPE1-GFP^C^. Full-length coding sequences of AaAtg1, AaAtg1^ΔFVFV^, and AaAtg1^AAVFAA^ were individually inserted into the pNEOGPE1-GFP^C^ vector to generate three plasmid constructs (Supplementary Table S2). Similarly, AaAtg8 and AaAtg13 coding sequences were individually cloned into pHygroGPE1-GFP^N^. The resulting plasmid pairs were co-transformed into wild-type protoplasts, and transformants were recovered on regeneration medium plates supplemented with 200 μg/ml hygromycin and 300 μg/ml geneticin. Transformants were confirmed by PCR. After incubation on PDA for 3 days, hyphae were examined microscopically for green fluorescence to evaluate GFP reconstitution.

### Toxin production and fungal virulence

Fungal strains were cultured in modified Richard’s medium [36] for 21 days, and culture filtrates were collected for ACT purification using Amberlite XAD-2 resin (Sigma-Aldrich). ACT was dissolved in methanol and analyzed by TLC and HPLC, as described previously [50]. Fungal pathogenicity was evaluated by applying 10 μl of conidial suspensions (1×10^5^ cells/ml) onto detached calamondin (*Citrofortunella mitis*) leaves, either unwounded or wounded with a fine needle. Leaves treated with sterile water served as a mock control. Treated leaves were incubated in a plastic box for 3–4 days to observe necrotic lesion development. Each fungal strain was tested on at least four leaves, and the experiments were repeated 3 times.

### Microscopy

Hyphae were stained with lactophenol-cotton blue (Sigma-Aldrich) in ethanol (4:1, v/v) for 20 min and observed using a Nikon Optiphot 2 Microscope (Tokyo, Japan). CMAC (7-amino-4-chloromethylcoumarin, 100 μM; Thermo Fisher Scientific, Waltham, MA, U.S.A.) was used to stain vacuoles. DCFHDA (2’-7’-dichlorofluorescein diacetate, Sigma-Aldrich) was used to detect cellular ROS. Cell viability was assessed using propidium iodide (PI, Sigma-Aldrich) staining. Fluorescence was detected using a ZOE Fluorescent Cell Imager (Bio-Rad) with appropriate excitation and emission wavelengths, as previously described [34, 42]. TEM was performed as described [42]. All experiments were repeated at least twice.

### Statistical analysis

Statistical analyses were performed using SPSS Statistics 20 software (IBM, NY, U.S.A.). Data are presented as means ± standard deviation. The homogeneity of variance was assessed with Levene’s test. Statistical significance was evaluated using either an independent sample *t*-test or one-way ANOVA, followed by Tukey’s honest significant difference (HSD) post hoc test (*p* < 0.05). Each treatment contained at least three replicates unless otherwise specified.

## Supplementary Material

This is a list of supplementary files associated with this preprint. Click to download.

• Supplementarymaterial.docx

• SupplementaryTablesS1andS2.docx

## Data Availability

The original contributions presented in the study are included in the article/Supplementary Material; further inquiries can be directed to the corresponding authors.

## Abbreviations

AaAtg: *Alternaria alternata* autophagy-related protein
AIM: Atg8-family-interacting motif
ROS: Reactive oxygen species
PDB: Potato dextrose broth
MM-N: Minimal medium without nitrogen
H_2_O_2_: Hydrogen peroxide
ACT: *Alternaria citri* tangerine toxin

## References

[1] Lahiri V, Hawkins WD, Klionsky DJ. Watch what you (self-) eat: autophagic mechanisms that modulate metabolism. Cell Metab. 2019;29(4):803–26. doi: 10.1016/j.cmet.2019.03.003. 30943392 PMC6450419

[2] Mizushima N, Yoshimori T, Ohsumi Y. The role of Atg proteins in autophagosome formation. Annu Rev Cell Dev Biol. 2011;27(1):107–32. doi: 10.1146/annurev-cellbio-092910-154005. 21801009

[3] Klionsky DJ, Ohsumi Y. Vacuolar import of proteins and organelles from the cytoplasm. Annu Rev Cell Dev Biol. 1999;15(1):1–32. doi: 10.1146/annurev.cellbio.15.1.1. 10611955

[4] Mizushima N. The role of the Atg1/ULK1 complex in autophagy regulation. Curr Opin Cell Biol. 2010;22(2):132–9. doi: 10.1016/j.ceb.2009.12.004. 20056399

[5] Noda NN, Fujioka Y. Atg1 family kinases in autophagy initiation. Cell Mol Life Sci. 2015;72:3083–96. doi: 10.1007/s00018-015-1917-z. 25948417 PMC4506457

[6] Suzuki K, Kubota Y, Sekito T, Ohsumi Y. Hierarchy of Atg proteins in pre-autophagosomal structure organization. Genes Cells. 2007;12(2):209–18. doi: 10.1111/j.1365-2443.2007.01050.x. 17295840

[7] Cheong H, Nair U, Geng J, Klionsky DJ. The Atg1 Kinase Complex Is Involved in the Regulation of Protein Recruitment to Initiate Sequestering Vesicle Formation for Nonspecific Autophagy in *Saccharomyces cerevisiae*. Mol Biol Cell. 2008;19(2):668–81. doi: 10.1091/mbc.e07-08-0826. 18077553 PMC2230592

[8] Kawamata T, Kamada Y, Kabeya Y, Sekito T, Ohsumi Y. Organization of the pre-autophagosomal structure responsible for autophagosome formation. Mol Biol Cell. 2008;19(5):2039–50. doi: 10.1091/mbc.e07-10-1048. 18287526 PMC2366851

[9] Stjepanovic G, Davies CW, Stanley RE, Ragusa MJ, Kim DJ, Hurley JH. Assembly and dynamics of the autophagy-initiating Atg1 complex. Proc Natl Acad Sci. 2014;111(35):12793–8. doi: 10.1073/pnas.1407214111. 25139988 PMC4156731

[10] Pollack JK, Harris SD, Marten MR. Autophagy in filamentous fungi. Fungal Genet Biol. 2009;46(1):1–8.doi: 10.1016/j.fgb.2008.10.010. 19010432

[11] Zhu X-M, Li L, Wu M, Liang S, Shi H-B, Liu X-H, Current opinions on autophagy in pathogenicity of fungi. Virulence. 2019;10(1):481–9. doi: 10.1080/21505594.2018.1551011. 30475080 PMC6550554

[12] Cai Y-Y, Li L, Zhu X-M, Lu J-P, Liu X-H, Lin F-C. The crucial role of the regulatory mechanism of the Atg1/ULK1 complex in fungi. Front Microbiol. 2022;13:1019543. doi: 10.3389/fmicb.2022.1019543. 36386635 PMC9643702

[13] Juarez-Montiel M, Clark-Flores D, Tesillo-Moreno P, de la Vega-Camarillo E, Andrade-Pavon D, Hernandez-Garcia JA, Vacuolar proteases and autophagy in phytopathogenic fungi: A review. Front Fungal Biol. 2022;3:948477. Epub 2023/09/25. doi: 10.3389/ffunb.2022.948477. 37746183 PMC10512327

[14] Noda T, Ohsumi Y. Tor, a phosphatidylinositol kinase homologue, controls autophagy in yeast. J. Biol. Chem. 1998;273(7):3963–6. doi: 10.1074/jbc.273.7.3963. 9461583

[15] Stephan JS, Yeh Y-Y, Ramachandran V, Deminoff SJ, Herman PK. The Tor and PKA signaling pathways independently target the Atg1/Atg13 protein kinase complex to control autophagy. Proc Natl Acad Sci. 2009;106(40):17049–54. doi: 10.1073/pnas.0903316106. 19805182 PMC2761351

[16] Meley D, Bauvy C, Houben-Weerts JH, Dubbelhuis PF, Helmond MT, Codogno P, AMP-activated protein kinase and the regulation of autophagic proteolysis. J Biol Chem. 2006;281(46):34870–9. doi: 10.1074/jbc.M605488200. 16990266

[17] Budovskaya YV, Stephan JS, Reggiori F, Klionsky DJ, Herman PK. The Ras/cAMP-dependent Protein Kinase Signaling Pathway Regulates an Early Step of the Autophagy Process in *Saccharomyces cerevisiae*. J Biol Chem. 2004;279(20):20663–71. doi: 10.1074/jbc.M400272200. 15016820 PMC1705971

[18] Schmelzle T, Beck T, Martin DE, Hall MN. Activation of the RAS/cyclic AMP pathway suppresses a TOR deficiency in yeast. Mol Cell Biol. 2004;24:338–51. doi: 10.1128/MCB.24.1.338-351.2004. 14673167 PMC303340

[19] Chang Y-Y, Neufeld TP. An Atg1/Atg13 complex with multiple roles in TOR-mediated autophagy regulation. Mol Biol Cell. 2009;20(7):2004–14. doi: 10.1091/mbc.e08-12-1250. 19225150 PMC2663935

[20] Puente C, Hendrickson RC, Jiang X. Nutrient-regulated phosphorylation of ATG13 inhibits starvation-induced autophagy. J Biol Chem. 2016;291(11):6026–35. doi: 10.1074/jbc.M115.689646. 26801615 PMC4786734

[21] Yao W, Li Y, Chen Y, Chen Y, Xie Y, Ye M, Atg1-mediated Atg11 phosphorylation is required for selective autophagy by regulating its association with receptor proteins. Autophagy. 2023;19(1):180–8. doi: 10.1080/15548627.2022.2063494. 35427192 PMC9809958

[22] Xie Z, Nair U, Klionsky DJ. Atg8 controls phagophore expansion during autophagosome formation. Mol Biol Cell. 2008;19(8):3290–8. doi: 10.1091/mbc.e07-12-1292. 18508918 PMC2488302

[23] Kraft C, Kijanska M, Kalie E, Siergiejuk E, Lee SS, Semplicio G, Binding of the Atg1/ULK1 kinase to the ubiquitin-like protein Atg8 regulates autophagy. EMBO J. 2012;31(18):3691–703. doi: 10.1038/emboj.2012.225. 22885598 PMC3442273

[24] Kabeya Y, Mizushima N, Ueno T, Yamamoto A, Kirisako T, Noda T, LC3, a mammalian homologue of yeast Apg8p, is localized in autophagosome membranes after processing. EMBO J. 2000;22:4577. doi: 10.1093/emboj/19.21.5720. PMC305793 11060023

[25] Kirisako T, Baba M, Ishihara N, Miyazawa K, Ohsumi M, Yoshimori T, Formation process of autophagosome is traced with Apg8/Aut7p in yeast. J Cell Biol. 1999;147(2):435–46. doi: 10.1083/jcb.147.2.435. 10525546 PMC2174223

[26] Nakatogawa H, Ichimura Y, Ohsumi Y. Atg8, a ubiquitin-like protein required for autophagosome formation, mediates membrane tethering and hemifusion. Cell. 2007;130(1):165–78. doi: 10.1016/j.cell.2007.05.021. 17632063

[27] Nakatogawa H, Ohbayashi S, Sakoh-Nakatogawa M, Kakuta S, Suzuki SW, Kirisako H, The autophagy-related protein kinase Atg1 interacts with the ubiquitin-like protein Atg8 via the Atg8 family interacting motif to facilitate autophagosome formation. J Biol Chem. 2012;287(34):28503–7. doi: 10.1074/jbc.C112.387514. 22778255 PMC3436563

[28] Jacomin A-C, Samavedam S, Promponas V, Nezis IP. iLIR database: A web resource for LIR motif-containing proteins in eukaryotes. Autophagy. 2016;12(10):1945–53. doi: 10.1080/15548627.2016.1207016. 27484196 PMC5079668

[29] Rogov VV, Nezis IP, Tsapras P, Zhang H, Dagdas Y, Noda NN, Atg8 family proteins, LIR/AIM motifs and other interaction modes. Autophagy Rep. 2023;2(1):2188523. doi: 10.1080/27694127.2023.2188523. 38214012 PMC7615515

[30] Fracchiolla D, Sawa-Makarska J, Martens S. Beyond Atg8 binding: The role of AIM/LIR motifs in autophagy. Autophagy. 2017;13(5):978–9. doi: 10.1080/15548627.2016.1277311. doi:10.1080/15548627.2016.1277311. 28121222 PMC5446069

[31] Thomma BP. Alternaria spp.: from general saprophyte to specific parasite. Mol Plant Pathol. 2003;4(4):225–36. doi: 10.1046/j.1364-3703.2003.00173.x. 20569383

[32] Meena M, Samal S. Alternaria host-specific (HSTs) toxins: An overview of chemical characterization, target sites, regulation and their toxic effects. Toxicol Rep. 2019;6:745–58. doi: 10.1016/j.toxrep.2019.06.021. 31406682 PMC6684332

[33] Wu P-C, Chen Y-K, Yago JI, Chung K-R. Peroxisomes implicated in the biosynthesis of siderophores and biotin, cell wall integrity, autophagy, and response to hydrogen peroxide in the citrus pathogenic fungus *Alternaria alternata*. Front Microbiol. 2021;12:645792. doi: 10.1111/mpp.13247. 34262533 PMC8273606

[34] Wu P-C, Choo CYL, Lu H-Y, Wei X-Y, Chen Y-K, Yago JI, Pexophagy is critical for fungal development, stress response, and virulence in *Alternaria alternata*. Mol Plant Pathol. 2022;23(10):1538–54. doi: 10.3389/fmicb.2021.645792. 35810316 PMC9452759

[35] Wu P-C, Choo Y-L, Wei S-Y, Yago JI, Chung K-R. Contribution of Autophagy to Cellular Iron Homeostasis and Stress Adaptation in *Alternaria alternata*. Int J Mol Sci. 2024;25(2):1123. doi: 10.3390/ijms25021123. 38256200 PMC10816921

[36] Kohmoto K, Itoh Y, Shimomura N, Kondoh Y, Otani H, Kodama M, Isolation and biological activities of two host-specific toxins from the tangerine pathotype of *Alternaria alternata*. Phytopathology. 1993;83(5):495–502. doi: 10.1094/PHYTO-83-495.

[37] Ma H, Zhang B, Gai Y, Sun X, Chung K-R, Li H. Cell-wall-degrading enzymes required for virulence in the host selective toxin-producing necrotroph *Alternaria alternata* of citrus. Front Microbiol. 2019;10:2514. doi: 10.3389/fmicb.2019.02514. 31824437 PMC6883767

[38] Lin C-H, Yang SL, Chung K-R. Cellular responses required for oxidative stress tolerance, colonization, and lesion formation by the necrotrophic fungus *Alternaria alternata* in citrus. Curr Microbiol. 2011;62:807–15. doi: 10.1007/s00284-010-9795-y. 20978890

[39] Chung K-R. Reactive oxygen species in the citrus fungal pathogen *Alternaria alternata*: The roles of NADPH-dependent oxidase. Physiol Mol Plant Pathol. 2014;88:10–7. doi: https://doi.org/10.1016/j.pmpp.2014.08.001. doi: 10.1016/j.pmpp.2014.08.001.

[40] Kabeya Y, Kamada Y, Baba M, Takikawa H, Sasaki M, Ohsumi Y. Atg17 functions in cooperation with Atg1 and Atg13 in yeast autophagy. Mol Biol Cell 2005;16(5):2544–53. doi:10.1091/mbc.E04-08-0669. 15743910 PMC1087256

[41] Titorenko VI, Rachubinski RA. Dynamics of peroxisome assembly and function. Trends Cell Biol. 2001;11(1):22–9. doi: 10.1016/s0962-8924(00)01865-1. 11146295

[42] Choo CYL, Wu P-C, Yago JI, Chung K-R. The Pex3-mediated peroxisome biogenesis plays a critical role in metabolic biosynthesis, stress response, and pathogenicity in *Alternaria alternata*. Microbiol Res.2023;266:127236. doi: 10.1016/j.micres.2022.127236. 36334316

[43] Lin C-H, Yang SL, Chung K-R. The YAP1 homolog–mediated oxidative stress tolerance is crucial for pathogenicity of the necrotrophic fungus *Alternaria alternata* in citrus. Mol Plant Microbe Interact. 2009;22(8):942–52. doi: 10.1007/s00284-010-9795-y. 19589070

[44] Chung K-R, Wu P-C, Chen Y-K, Yago JI. The siderophore repressor SreA maintains growth, hydrogen peroxide resistance, and cell wall integrity in the phytopathogenic fungus *Alternaria alternata*. Fungal Genet Biol 2020;139:103384. doi: 10.1016/j.fgb.2020.103384. 32278718

[45] Mulder NJ, Apweiler R. The InterPro Database and Tools for Protein Domain Analysis. Curr Protoc Bioinform. 2008;21(1):2.7.1–2.7.18. doi: 10.1002/0471250953.bi0207s21. 18428686

[46] Liu W, Xie Y, Ma J, Luo X, Nie P, Zuo Z, IBS: an illustrator for the presentation and visualization of biological sequences. Bioinformatics. 2015;31(20):3359–61. doi: 10.1093/bioinformatics/btv362. 26069263 PMC4595897

[47] Mirdita M, Schütze K, Moriwaki Y, Heo L, Ovchinnikov S, Steinegger M. ColabFold: making protein folding accessible to all. Nat Methods. 2022;19(6):679–82. doi: 10.1038/s41592-022-01488-1. 35637307 PMC9184281

[48] Cung K-R, Shilts T, Li W, Timmer LW. Engineering a genetic transformation system for *Colletotrichum acutatum*, the causal fungus of lime anthracnose and postbloom fruit drop of citrus. FEMS Microbiol Lett. 2002;213(1):33–9. doi: 10.1111/j.1574-6968.2002.tb11282.x. 12127485

[49] Jenns A, Daub M, Upchurch R. Regulation of cercosporin accumulation in culture by medium and temperature manipulation. Phytopathology. 1989;79(2):213–9. doi: 10.1094/Phyto-79-213.

[50] Wu P-C, Chen C-W, Choo CYL, Chen Y-K, Yago JI, Chung K-R. Proper functions of peroxisomes are vital for pathogenesis of citrus brown spot disease caused by *Alternaria alternata*. J Fungi. 2020;6(4):248. doi: 10.3390/jof6040248 PMC7712655 33114679

